# Knowledge, attitude and practice level of women at the periconceptional period: a cross-sectional study in Shaanxi China

**DOI:** 10.1186/s12884-019-2481-6

**Published:** 2019-09-04

**Authors:** Danyang Li, Liyan Huang, Wenfang Yang, Cuifang Qi, Li Shang, Juan Xin, Lingxia Zeng, Min Zhang, Hui Song, Mei Chun Chung

**Affiliations:** 1 Department of Obstetrics and Gynecology, The First Affiliated Hospital of Xi’an Jiaotong University, Xi’an, Shaanxi People’s Republic of China; 2 Department of Public Health and Community Medicine, Tufts University School of Medicine, Boston, Massachusetts USA; 3 Department of Epidemiology and Biostatistics, School of Public Health, Xi’an Jiaotong University Health Science Center, Xi’an, Shaanxi People’s Republic of China; 4 Maternal and Child Health Hospital of Xi’an, Xi’an, Shaanxi People’s Republic of China

**Keywords:** KAP level, Periconceptional period, Cross-sectional study, Shaanxi China

## Abstract

**Background:**

Identifying and understanding the knowledge, attitude and practice (KAP) level of women at the periconceptional period has implications for formulating and measuring the adverse pregnancy outcomes for primary prevention.

**Methods:**

A cross-sectional study among pregestational and pregnant women was conducted in Shaanxi during 2016–2017.

**Results:**

Among 791 participants, the average score of periconceptional healthcare knowledge awareness was 6.32 ± 1.78, whereas 28.8% of women have failed. Women who planned to or had undergone premarital and pre-pregnancy examinations accounted for 50.2, and 62.5%, respectively. Less than half (42.0%) of the women started taking folic acid (FA) before pregnancy, and only 37.9% of them took FA regularly at the right time. Multivariate analysis showed that age was the main factor influencing the Attitude and Practice level of women at the periconceptional period, and demonstrated a positive effect on the awareness of right timing of folic acid supplementation, and high rates of premarital and pre-pregnancy examinations. Also, the knowledge pass rate was increased with education level. Fewer women who have birth experience were willing to take FA consistently at the right time compared to those women without birth.

**Conclusions:**

The women at the periconceptional period in Shaanxi lacked the total KAP level of periconceptional healthcare, especially those who live in rural areas and have less education. Government agencies should reinforce more effective primary preventive measures and policies for the prevention of adverse pregnancy outcomes.

**Electronic supplementary material:**

The online version of this article (10.1186/s12884-019-2481-6) contains supplementary material, which is available to authorized users.

## Background

Adverse pregnancy outcomes are significant health challenges for women in the periconceptional period as well as infants. These include pregnancy bleeding, hypertensive disorders and sepsis, which lead to more than half of the maternal deaths [1]. It is estimated that about 14.9 million preterm infants are born every year, accounting for 11.1% live births, and more than half of them are born in Asian and African countries including China [2]. The mortality rate of infants and children under the age of 5 in China was significantly lower in eastern and urban areas compared with mid-western and rural areas [3]. A cross-sectional survey of a large sample of Shaanxi Province revealed that the incidence of adverse pregnancy outcomes was 25.45%, which was higher than the national average level [4]. According to the government data on birth defects in the Shaanxi province, the prevalence in the last ten years showed a steady rate and did not decline with time (Additional file 1: Table S1). After several years of government efforts to promote folic acid (FA) supplementation in pregnant women, the prevalence of neural tube defects (NTDs) in China has been decreased from 11.40‰ in 2000 to 5.74‰ in 2010 and 2.81‰ in 2014, and its rank in birth defects remained stable in the top 10 [5]. The NTDs are also considered as the main reason for birth defects in developed countries [6].

Therefore, it is necessary to adopt preventive measures to control and relieve the occurrence of these diseases. Primary prevention including specific practices of health promotion, protective procedures, detection and regulation of environmental pollutants is considered as an essential way for adverse pregnancy outcomes remission. Previous studies have demonstrated that about 70% of adverse pregnancy outcomes such as birth defects are effectively prevented or cured with proper care [7]. One critical step for the primary prevention practice is to investigate the knowledge, attitude and practice (KAP), which mainly evaluates the understanding and grasping of knowledge, attitude and expectation as well as related behaviours of the participants. It is confirmed that KAP interacted with each other. This survey was conducted based on the principle that increasing knowledge results in changing attitudes and practices to minimise disease burden [8]. Some practices can be accepted before knowledge increase and attitude change to alter people’s awareness of diseases and their physical condition and reduce the number of diseases and social burden [9]. Understanding the KAP level remains helpful to identify the knowledge gap and determine behaviours of the public to further plan and implement for the prevention of adverse pregnancy outcomes.

Scientific evidence has demonstrated that some risk factors and protective factors such as FA [10] and micronutrient supplementation [11] were correlated with adverse. FA supplementation during the periconceptional period has been started to popularise since the 1990s in China to reduce adverse pregnancy outcomes, especially the birth defects. According to a previous study that focused on the preventive knowledge of FA in Shanghai, more than half of the people did not understand the role of FA, and only one-third of the women knew the right time for taking FA [12]. However, in Israel and America, the number of women with the knowledge of FA roles (80.8% & 74%) and the right time to take folic acid supplements (74.6% & 55%) are higher than Shanghai [13, 14]. The rate of FA regular usage in Shaanxi (11.2%) was more upper than Korea (10.3%) but lower than Japan (85%) [15–17].

Also, premarital, pre-pregnancy and prenatal examinations are the three routine inspections recommended for women at the periconceptional period to ensure the health of both mothers as well as children and the examination results are directly related to the adverse pregnancy outcomes [18]. In China, the number of women at the periconceptional period undertaking routine examinations and participation in the awareness programs of pre-pregnancy health in public is still quite limited compared to developed countries [19].

Since 2009, the provincial government of Shaanxi decided to give away free FA supplements (0.4 mg/d) to all the women at the periconceptional period through the Family Planning System and Maternal and Child Health Care System. At the same time, the government switched their attention from secondary and tertiary prevention to primary prevention. After the implementation of government policies, attention has been paid on understanding and actions of primary prevention of adverse pregnancy outcomes in public, especially in women at the childbearing age. It is necessary to investigate the attitudes and behaviours of the society about the current policies as well as to provide evidence for the local government for formulating the priority targets and providing recommendations on the current actions regarding the primary prevention in Shaanxi. Hence, a cross-sectional study aiming at women at the periconceptional period was conducted in 2016–2017 to investigate the women’s eugenic KAP level on adverse pregnancy outcomes as primary prevention.

## Material and methods

### Study objects

From October 2015 to February 2017, 791 women were gathered in Yan ‘an and Xi ‘an, Shaanxi province, respectively. Eligible women were who prepared to pregnant in 3 months or at the duration of pregnancy, and lived in the local area for more than 1 year. For the participants from Yan’an, a stratified, random sampling method was used. Women who met the requirements were randomly selected from four counties (Baota, Ansai, Yanchang, Ganquan) out of thirteen counties. They were approached through the periconceptional healthcare lectures that we conducted in each selected county, which assisted by the Yan’an Health Committee. For participants in Xi’an, they were randomly selected from women who attended physical examinations in the First Affiliated Hospital of Xi’an Jiaotong University. Women were identified by asking the question that “Are you now in pregnant?” and “Do you have any plans for pregnancy in the latest three months?” into pre-pregnant and pregnant groups. The informed consent form was obtained from each participant and their phone numbers and residential addresses were recorded.

### Study content and survey methods

Women were investigated and questioned by one-to-one interviews. The questionnaire was formulated by experts of family planning. All questions assessed the KAP level of women at the periconceptional period regarding periconceptional healthcare, including basic social demographic characteristics of the participants (age, location, education background for women and their husbands, average monthly family income, and pregnancy history); knowledge level on periconceptional health care (Knowledge section); the participation and demands of pre-pregnancy and prenatal examination (Attitude section), and FA taking compliance (Practice section).

The survey of KAP three sections has been completed at the time of recruitment for women who have been pregnant for more than 3 months. For women who were preparing for pregnancy and who have been pregnant but less than 3 months, only two parts of the survey, Knowledge and Attitude sections, have been completed at the time of recruitment. A Follow-up survey was conducted to investigate folic acid use during the 3 months before pregnancy (at the first prenatal examination) and the first trimester of pregnancy (at the 13–16^+ 6^ weeks’ prenatal examination). Finally, A total of 791 valid questionnaires were obtained for the survey of Knowledge and Attitudes sections, of which 643 were obtained for complete folic acid supplementation information, due to 97 (12.23%) women who were preparing for pregnancy but were not pregnant, 51 (6.45%) women were lost to follow-up during pregnancy.

In terms of Knowledge part, 10 closed choice questions were designed aiming at the essential knowledge points of periconceptional healthcare such as the concept, and categories of birth defects, FA supplementation time and the importance of prenatal examination (details are shown in Additional file 2: Table S2). Except for the question “classification of birth defects” was multiple-choice, the remaining nine were single-choice questions. For the “classification of birth defects”, we listed four most common birth defects for choices: anencephaly, hydrocephalus, cleft lip and palate, and congenital heart disease, and asked women to tick off the conditions they considered as birth defects. It was considered as classified correctly if all four options had been selected, missing or not selected were classified incorrectly. For the remaining nine single-choice questions, only one of the options was correct. For example, the question in terms of “the importance of prenatal examinations” was that “All my examinations before pregnancy were normal, do I need to have prenatal examinations?” The answer was “Yes, because the pre-pregnancy examinations cannot take the place of prenatal examinations”, and the rest of the options were “No, because many items have been checked before pregnancy, it is enough to guarantee a healthy baby”; “Not necessarily required, prenatal examination is only necessary if there is a problem in pre-pregnancy check” and “Have no idea”. Closed quantify scoring system was used for the 10 questions, where 1 question corresponded to 1 point. When the score was higher than or equal to 6, it was defined as knowledge pass.

For the attitude level, the participation rate of the pre-pregnancy and premarital examination was investigated as the women’s attitude of periconceptional healthcare. Participation of regular examinations in this research was defined as women who planned to participate or had participated in the premarital examination and pre-pregnancy examination. The practice part was represented by FA taking. “FA taking every day” was defined as taking pills containing at least 0.4 mg FA every day without interruption during 3 months before pregnancy or the first trimester of pregnancy.

### Statistical analysis

After data cleaning and quality check, all data were coded, and Epidata3.1 was used for data entry and logic error detection. Quantitative data were described as mean ± standard deviation, and categorical data were presented by rates or composition ratio. In terms of knowledge related to periconceptional health care, there were 10 questions in total. Each received one point for the correct answer of each item, and the score ranged from 0 to 10 points. After averaging the scores of all participants, the overall average score of the knowledge level was obtained. Presentation of all tables was executed by using Microsoft Excel 2010. Differences in the knowledge level, participation rate of regular examinations and FA taking were used as dependent variables to represent KAP level. Different demographic characteristics, such as women’s region, age, location, educational background, and family income, were regarded as independent variables. Bivariate analysis such as ANOVA and χ^2^ test and multivariate logistic regression analysis were used to evaluate the influence of social demographic characteristics on KAP level. All data were analyzed by SPSS 20.0. *P* <  0.05 was considered as statistical significance.

### Ethical statement

This research was approved by the Ethics Committee of Science of Medical center, The First Affiliated Hospital of Xi’an Jiaotong University (No:XJTU1AF2017LSK-45). The written informed consent form was obtained from each participant who took part in this survey.

## Results

### Basic characteristics of women at the periconceptional period

A total of 791 women at the periconceptional period finished our interview, which included 451 from Yan’an (located in the north of Shaanxi) and 340 from Xi’an (located in the middle part of Shaanxi). Among these interviews, the average age of the women was 28.42 ± 3.99 (with a maximum of 45 and a minimum of 19). The main age group was 26–30 years old, which accounted for 56.1%. The proportion of women living in city and suburb/rural areas was 72.0 and 28.0%, respectively. In all families, almost half of the women and their husbands were graduated from senior high school/college, and about 35.3% of them were undergraduates or above. Only 19.7% women and 21.3% of husbands were from junior high school or below. The family income ranged from 4000 to 12,000 RMB, accounting for 53.9%. 16.9% of women preparing for pregnancy, while 83.1% of them already had pregnancy during the investigation time, and 69.6% of them had no pregnancy experience at all (Table 1).

**Table 1:** KAP overview and bivariate analysis

	Total		Knowledge Pass ^a^		Attitude				Practice			
					Premarital examination ^b^		Pre-pregnancy examination ^c^		FA taking Pre-pregnancy ^d^		FA taking everyday ^e^	
	N	%	N	%	N	%	N	%	N	%	N	%
Total	791	100	563	71.2	397	50.2	494	62.5	270	42.0	527	81.6
Region												
Yan an	451	57.02	237	57.8	284	63.0	337	75.6	99	32.2	212	69.1
Xi’an	340	42.98	276	83.4	113	33.2	157	46.2	171	50.9	315	92.9
χ^2^	–		56.25		68.57		71.34		22.90		61.06	
P	–		< 0.001*		< 0.001*		< 0.001*		< 0.001*		< 0.001*	
Age												
≤ 25	158	20.2	78	54.2	76	48.1	95	60.1	30	25.0	88	73.9
26–30	440	56.1	298	72.2	231	52.5	280	64.1	182	48.8	315	83.8
31–35	141	18.0	99	74.4	64	45.4	89	63.1	46	39.0	99	83.2
> 35	45	5.7	35	77.8	22	48.9	27	62.8	11	39.3	24	85.7
χ^2^	–		20.28		2.54		0.78		21.81		6.39	
P	–		< 0.001*		0.47		0.86		< 0.001*		0.09	
Location												
City	568	72.0	393	73.3	276	48.6	354	62.7	209	44.7	391	83.2
Suburb/rural area	221	28.0	120	59.1	120	54.3	139	63.5	61	34.9	136	77.3
χ^2^	–		14.00		2.07		0.05		5.02		2.99	
P	–		< 0.001*		0.15		0.87		0.03*		0.08	
Education												
Junior high school or below	155	19.7	61	44.2	81	52.3	101	65.6	26	22.2	73	61.9
Senior high school/college	354	45.0	228	67.5	187	52.8	221	62.8	127	43.3	246	84.2
Undergraduate or above	278	35.3	221	84.4	128	46.0	171	62.0	117	51.1	205	88.4
χ^2^	–		69.04		3.16		0.58		26.72		39.07	
P	–		< 0.001*		0.21		0.75		< 0.001*		< 0.001*	
Husband education												
Junior high school or below	167	21.3	76	50.7	90	53.9	113	67.7	31	23.5	90	67.7
Senior high school/college	334	42.8	212	66.9	170	50.9	202	60.8	120	43.3	230	83.0
Undergraduate or above	280	35.9	222	83.1	134	47.9	174	62.6	117	51.5	203	88.6
χ^2^	–		49.57		1.57		2.24		27.23		25.38	
P	–		< 0.001*		0.46		0.33		< 0.001*		< 0.001*	
Family income (per month)												
≤ 4000	303	39.1	148	53.2	169	55.8	212	70.4	66	28.9	157	68.9
4000–12,000	417	53.9	312	78.4	199	47.7	243	58.7	169	48.4	313	89.2
> 12,000	54	7.0	44	84.6	21	38.9	27	50.0	28	53.8	47	90.4
χ^2^	–		54.84		7.56		14.25		24.88		41.27	
P	–		< 0.001*		0.02*		0.001*		< 0.001*		< 0.001*	
Pregnancy history												
No	550	69.6	358	69.0	270	49.1	342	62.4	217	46.2	400	84.4
Yes	240	30.4	155	69.8	126	52.5	151	63.7	53	30.6	127	73.8
χ^2^	–		6.05		0.78		2.19		12.53		9.35	
P	–		0.015*		0.40		0.14		< 0.001*		0.002*	

*:*P* <  0.05

^a^ Women whose scores on the assessment of knowledge level were higher than or equal to 6 (*n* = 791)

^b^ Women who planned to participate or had participated in premarital examinations (*n* = 791)

^c^ Women who planned to participate or had participated in pre-pregnancy examinations (*n* = 791)

^d^ women who started taking FA before pregnancy (*n* = 643)

^e^ women who have taken pills containing at least 0.4 mg FA every day without interruption during 3 months before pregnancy or the first trimester of pregnancy (*n* = 643)

### Knowledge level and bivariate analysis

The results from Table 2 indicated that the average knowledge level score of 791 women was 6.32 ± 1.78, wherein 28.8% of women failed, and their average score was below 6 points. Most of the women acquired 7 points (20.4%), but only 10 women got full score. Besides, the importance of prenatal examination showed the highest awareness according to these 10 knowledge points, reaching 90.1%. Most of them lacked knowledge of the dosage of folate intake (8.6%).

**Table 2:** Bivariate analysis of knowledge level

	Average score	Pass rate	Birth defects	Birth defects classification	Primary prevention	Neonatal screening	Men’s pre-pregnancy check	FA supplementary time	FA supplemetary dosage	Importance of prenatal examination	Advanced pregnancy	Thyroid dysfunction and eugenics
Total	6.32 ± 1.78	71.2	82.9	72.2	67.3	85.8	68.4	46.4	8.6	90.1	63.3	67.5
Age												
≤ 25	5.48 ± 1.74	54.2	84.2	88.4	68.9	87.7	56.1	31.6	3.8	84.8	44.9	58.6
26–30	6.40 ± 1.71	72.2	85.4	70.6	70.1	88.2	73.1	49.0	10.2	92.9	63.0	69.7
31–35	6.78 ± 1.67	74.4	85.9	66.7	65.2	85.9	72.6	61.5	6.7	96.3	84.3	75.4
> 35	6.96 ± 1.83	77.8	84.4	73.3	75.6	88.9	71.1	48.9	20.0	95.6	84.4	77.8
F /χ^2^	13.46	20.28	0.49	23.64	2.04	0.57	16.40	26.66	13.45	15.55	57.47	12.20
P	< 0.001*	< 0.001*	0.98	< 0.001*	0.56	0.90	< 0.001*	< 0.001*	0.004*	0.001*	< 0.001*	0.007*
Location												
City	6.54 ± 1.71	73.3	86.5	72.1	70.8	88.7	72.3	52.6	10.7	93.4	67.0	70.4
Suburb/rural	5.74 ± 1.81	59.1	81.7	76.4	65.7	85.1	63.4	34.1	3.7	87.6	58.1	65.4
t/χ^2^	31.36	14.00	2.80	1.44	1.85	1.86	5.78	21.20	9.44	7.01	5.41	1.78
P	< 0.001*	< 0.001*	0.11	0.24	0.19	0.17	0.018*	< 0.001*	0.002*	0.008*	0.024*	0.20
Education												
Junior highschool or below	5.14 ± 1.73	44.2	83.9	86.2	67.8	79.5	46.7	27.5	5.3	77.9	51.3	53.3
Senior high school/college	6.21 ± 1.69	67.5	82.4	71.1	71.8	87.6	69.0	43.6	7.1	94.0	64.2	66.5
Undergraduate or above	7.06 ± 1.55	84.4	90.0	70.0	68.1	92.3	83.2	62.6	12.2	96.7	71.7	80.1
F/χ^2^	62.56	69.04	7.41	15.42	1.25	14.75	61.39	51.13	7.58	49.80	17.81	33.87
P	< 0.001*	< 0.001*	0.025*	< 0.001*	0.54	0.001*	< 0.001*	< 0.001*	0.023*	< 0.001*	< 0.001*	< 0.001*
Husband education												
Junior high school or below	5.31 ± 1.765	50.7	84.4	86.5	70.1	83.4	52.4	24.8	7.3	83.2	44.9	55.8
Senior high school/college	6.28 ± 1.745	66.9	84.1	71.2	67.5	88.4	67.3	49.7	6.1	93.9	66.6	66.7
Undergraduate or above	6.96 ± 1.506	83.1	87.6	68.5	72.3	90.2	83.3	58.0	12.4	94.9	73.1	79.1
F/χ^2^	47.29	49.57	1.69	18.60	1.62	4.56	48.16	45.91	8.11	22.06	37.30	27.30
P	< 0.001*	< 0.001*	0.43	< 0.001*	0.45	0.10	< 0.001*	< 0.001*	0.017*	< 0.001*	< 0.001*	< 0.001*
Family income (per month)												
≤ 4000	5.51 ± 1.662	53.2	83.7	89.9	68.9	82.4	60.3	27.7	4.1	84.7	53.0	60.4
4001–12,000	6.76 ± 1.638	78.4	86.3	65.5	69.5	90.5	74.9	58.6	10.5	96.3	72.0	73.7
> 12,000	7.10 ± 1.774	84.6	84.9	51.9	67.9	96.3	75.9	63.0	17.0	94.3	77.4	79.2
F/χ^2^	52.31	54.84	0.92	68.79	0.06	14.33	18.64	72.13	14.61	31.17	31.20	17.01
P	< 0.001*	< 0.001*	0.63	< 0.001*	0.97	0.001*	< 0.001*	< 0.001*	0.001*	< 0.001*	< 0.001*	< 0.001*
Pregancy history												
No	6.29 ± 1.785	69.0	83.1	72.0	70.3	88.1	69.4	49.7	8.5	91.5	62.2	69.1
Yes	6.38 ± 1.762	69.8	90.0	76.6	67.5	86.3	70.5	41.8	9.4	92.3	69.4	68.4
t/χ^2^	0.45	0.05	6.05	1.77	0.57	0.50	0.10	4.08	0.18	0.15	3.62	0.04
P	0.50	0.86	0.015*	0.22	0.49	0.48	0.80	0.043*	0.68	0.78	0.06	0.87

*P < 0.05

With increasing age of the women, the rate of knowledge transfer and the common knowledge were increased obviously among the participants. Women aged > 35 obtained the highest average score (6.96 ± 1.83), women < 25 years acquired the least average score (5.48 ± 1.74). In addition to the concept of birth defects, primary prevention, FA supplementary time and dosage, and neonatal screening showed significant differences in the average knowledge and overall knowledge passing rate among women of different ages. The average score and passing rate of women living in suburb/rural areas were significantly lower than those women living in cities, especially for women’s pre-pregnancy examination, FA supplementary time and dosage, the importance of prenatal examination as well as high-risk pregnancy. With the improvement of the educational background of both women and their husbands, the knowledge level was promoted as well. Except for the concept of primary prevention, the passing rates of other questions were also increased significantly in women with higher education. Moreover, increased family income facilitated women’s knowledge level apart from birth defects and primary prevention. However, pregnancy history showed no influence on the overall knowledge level of women at the periconceptional period, and the results were shown in Table 2.

### Attitude and practice level and bivariate analysis

In terms of regular examinations, the proportion of 791 women who planned to participate or had participated in the premarital examination and pre-pregnancy check were 50.2 and 62.5%, respectively. The participation rate in the premarital examination was significantly lower than the pre-pregnancy check (χ^2^ = 221.215, *P* <  0.001). In terms of FA use, majority of the women (94.2%) took FA during their pregestational and gestational periods, but less than half (42.0%) of the women started taking FA before pregnancy. There were 81.6% of women who insist on taking FA every day. However, only 37.9% of participants took FA regularly at the right time (Table 1).

From Table 2, it could be seen that the increase of family income significantly reduced the participation of premarital and pre-pregnancy examinations. Meanwhile, in terms of the right time to start taking folic acid, different factors including women’s age, location, education of both women and their husband, and family income demonstrated apparent effects on FA use. More women who were aged 26–30, living in the city and had no birth took FA at the right time. Also, women and their husbands with higher educational background were more likely to take FA at the right time and insist on taking every day. The increase of family income promoted FA insistence. Compared with women who gave birth, women without any history of pregnancy insisted on taking FA every day.

### Multivariate analysis of KAP

From Table 3, it could be seen that age was the main factor that influenced the Attitude and Practice level of women during the periconceptional period. Compared to women aged < 25 years, more women of 26–30 years were willing to take FA in the right time (OR:2.21, 95%CI:1.33–3.70) and insist on taking every day (OR:2.30, 95%CI:1.34–3.96), and were positively associated with high rates of premarital (OR:1.82,95%CI:1.12–2.81) and pre-pregnancy examinations (OR:2.03, 95%CI: 1.29–3.21). Besides, the knowledge pass rate was increased with education level whereas the significant association was only shown for senior high school or college category (OR: 1.69, 95%:1.02–2.78). However, a negative association was found between women with birth experience and intake of FA consistently at the right time.

**Table 3:** Multivariate analysis of KAP

	Knowledge pass ^a^		Attitude				Practice			
			Premarital examination ^b^		Pre-pregnancy examination ^c^		FA taking pre-pregnancy ^d^ Pre-pregnancy d Pre-pregnancy d		FA taking everyday ^e^	
	OR	95% CI	OR	95% CI	OR	95% CI	OR	95% CI	OR	95% CI
Age										
≤ 25	1.00	–	1.00	–	1.00	–	1.00	–	1.00	–
26–30	1.10	0.69, 1.74	1.82	1.12, 2.81*	2.03	1.29, 3.21*	1.82	1.12, 2.81*	2.30	1.34, 3.96*
31–35	1.02	0.53, 1.92	1.62	0.91, 2.86	2.70	1.48, 4.93	1.62	0.91, 2.86	1.57	0.79, 3.12
> 35	1.33	0.51,3.42	1.94	0.85, 4.41	3.30	1.38, 7.88	1.94	0.85, 4.41	2.16	0.78, 5.94
Region										
Yan an	1.00	–	1.00	–	1.00	–	1.00	–	1.00	–
Xi’an	4.52	2.90,7.06*	0.37	0.24,0.58*	0.20	0.12,0.33*	3.09	2.07,4.60*	5.91	3.32,10.51*
Location										
City	0.99	0.66, 1.51	0.78	0.54, 1.14	0.95	0.64, 140	0.78	0.54, 1.14	0.81	0.47, 1.38
Suburb/rural area	1.00	–	1.00	–	1.00	–	1.00	–	1.00	–
Education										
Junior high school or below	1.00	–	1.00	–	1.00	–	1.00	–	1.00	–
Senior high school/college	1.69	1.02, 2.78*	1.15	0.71, 1.89	1.07	0.63, 1.81	1.15	0.71, 1.89	1.23	0.66, 2.28
Undergraduate or above	3.19	1.61, 6.04*	0.96	0.53, 1.75	1.14	0.60, 2.17	0.96	0.53, 1.75	1.32	0.64, 2.73
Husband education										
Junior high school or below	1.00	–	1.00	–	1.00	–	1.00	–	1.00	–
Senior high school/college	1.13	0.69,1.84	1.16	0.72, 1.87	0.97	0.58, 1.63	1.16	0.72, 1.87	1.45	0.80, 2.61
Undergraduate or above	1.55	0.84, 2,89	1.28	0.71, 2.28	1.28	0.69, 2.40	1.28	0.71, 2.28	1.34	0.67, 269
Family income (per month)										
≤ 4000	1.00	–	1.00	–	1.00	–	1.00	–	1.00	–
4001–12,000	1.37	0.89,2.09	1.23	0.82,1.83	0.88	0.57,1.35	1.23	0.82,1.83	1.49	0.93, 2.38
> 12,000	1.31	0.52, 3.26	1.38	0.66,2.84	0.81	0.39,1.68	1.38	0.66,2.84	1.56	0.72,3.37
Pregnancy history										
No	1.00	–	1.00	–	1.00	–	1.00	–	1.00	–
Yes	1.31	0.84, 2.05	0.94	0.40, 1.39	0.68	0.45, 1.02	0.54	0.35,0.84*	0.52	0.33, 0.82*

*P < 0.05

^a^ Women whose scores on the assessment of knowledge level were higher than or equal to 6. (*n* = 791)

^b^ Women who planned to participate or had participated in premarital examinations. (*n* = 791)

^c^ Women who planned to participate or had participated in pre-pregnancy examinations. (*n* = 791)

^d^ women who started taking FA before pregnancy. (*n* = 643)

^e^ women who have taken pills containing at least 0.4 mg FA every day without interruption during 3 months before pregnancy or the first trimester of pregnancy. (*n* = 643)

## Discussion

Birth defects are influenced by different factors. Among these aspects, KAP is an integral part of primary prevention of birth defects. The attitude and practice of premarital and pre-pregnancy examinations, dietary habits, and drug intake of maternal women could affect their pregnancy outcomes. Moreover, KAP is not only an effective way to assess women’s knowledge and behavior during pregnancy but also an essential means to promote and improve primary prevention measures. In this study, the knowledge level of the women at the childbearing age showed a lack of awareness in the total knowledge. The average score was lower than that of a previous survey conducted in Shaanxi, 2006 [20]. Besides, the knowledge level was significantly different in women of different ages, educational level, and location. It was proved that younger women from the rural area with a lower educational level and income more likely lacked knowledge on periconceptional healthcare. Also, in Shaanxi, Zhang found that pregnant women who had better education demonstrated higher awareness of prenatal care and birth defects prevention [18]. Therefore, combined with the previous studies, it was concluded that women at the periconceptional period have more cognition on prenatal care, but little improvement on preventive measures, especially taking FA during pre- and early-pregnancy was performed. Hence, several measures should be taken to strengthen the primary prevention knowledge, especially for rural areas, poor education and low-income women for promoting the understandability and multi-way health promotion education for improving the level of eugenic knowledge. These prevention measures should be particularly taken 3 months before pregnancy and the organogenesis during the pregnancy (week 3–8 of pregnancy for the CNS) for preventing birth defects [21].

In this study, the majority of women at the periconceptional period took FA during pregnancy, but less than half (37.9%) took FA before pregnancy, and this was similar to the previous FA intake rate in Oregon, USA (33.2%) [22], but was lower than Tianjin (40.4%) located in the northeast of China [23]. In addition, 81.6% of women insisted on taking FA, but only 37.9% of them took FA regularly for 3 months before pregnancy. So, it was concluded that most women are clear regarding the importance of FA and are willing to take FA; however, they had an insufficient understanding of how to take FA. Furthermore, women tended to take FA regularly if they were older, living in urban areas or had no children, and higher education. Similar results were also found in America and Denmark [24, 25]. Tamim also found that women who had less number of pregnancies took FA more regularly [26]. In this study, it was suggested that lower correct rate of FA supplementation (including starting time and supplementary dose) was consistent with lower knowledge awareness of FA supplementation, which was in agreement with other previous studies [15, 26]. Kim et al. also showed that lower awareness on the effect of FA reduced the behavior of FA proper intake [15], and hence it was necessary to impart FA knowledge and benefits through health education or promotion, influencing the attitude and behavior of women during childbearing age. Other studies also demonstrated that planning for pregnancy had an impact on prenatal care, including maternal FA awareness, usage, and nutritional supplements [27, 28]. Hence, more specific FA intake knowledge and methods, especially the importance of pre-pregnancy FA usage, should be reinforced to the public with low education, low income and rural women.

As a protective factor, FA plays a crucial role in adverse pregnancy outcomes such as NTD and CHD prevention [10, 29]. However, low KAP level regarding the proper use of FA in women of Shaanxi may be a potential risk factor for causing these diseases, and similar results were found in the US [30]. Besides, the previous study found that the average rate of birth defects in rural areas was higher than that in urban areas in Xi’an [29]. In our study, women from rural areas and with poor education and family income tended to have lower KAP level, indicating that more attention should be paid on them to reduce the occurrence of adverse pregnancy outcomes. Nevertheless, our investigation on women at the childbearing age was just a cross-sectional study, and so the possible pregnancy outcomes were not obtained in the end. Whether the surveyed women with different KAP levels would directly influence the pregnancy outcomes still needs confirmation in the future.

The rate of premarital and pre-pregnancy examinations in our study was 50.2 and 62.5%, respectively, which was still lower compared with other developed countries [19]. The three most common reasons for not participating in these examinations include “have done a physical examination, so don’t need to undergo these examinations”, “all families are healthy, so does not require these examinations”, and “do not know how to undergo these examinations”. It indicated that women at the childbearing age lacked awareness regarding the significance and importance of these examinations and prevention of pregnancy disorders. In China, since the mandatory premarital examination was abolished in 2003, the importance of regular check, especially before marriage and pregnancy examinations, has been diminished in public, followed by the concomitant declination of premarital examinations [31]. Studies in Zhejiang and Jiangsu showed that the proportion of pre-marital records was decreased from 71.3 to 14.6%, and the rate of early pregnancy examinations was reduced from 64.0 to 50.0%. Moreover, it was considered that with the increased popularity of public physical examinations in recent years, the rate of participation in these eugenic examinations was reduced. In terms of influential factors, it was found that the educational levels were related to the participation rate of premarital and pre-pregnancy examinations [18]. In consequence, more effective and comprehensive publicity should be carried out to focus on the necessity and importance of regular examinations of premarital and pre-pregnant periods as well as the differences between these checks and public physical examinations for women of childbearing age.

We need to acknowledge some limitations of this study. First of all, the loss of information was unavoidable during the follow-up of some participants because of changes in their phone number or residential address. In addition, we surveyed the KAP levels of 791 women at the periconceptional period in total, but some of their basic characteristic data were incomplete (e.g. the number of data obtained on age was 784), which resulted in some loss of information from the bivariate and multivariate analyses. Secondly, some of our respondents were enrolled from the hospitals, although the method of random sampling was adopted, the choice bias was challenging to avoid compared with the community population. Thirdly, the recall bias could not be eliminated completely due to limitations in the cross-sectional design, although a series of practical measures were used. Finally, while we included several important confounding factors, we cannot rule out potential residual confounding. For instance, we did not have access to individual characteristics such as ethnicity, floating population, and health insurance that may affect the outcomes.

## Additional files

Additional file 1: **Table S1.** Birth defects in Xi’an from 2003 to 2016. (DOCX 21 kb)

Additional file 2: **Table S2.** Ten closed choice questions to assess knowledge level section^**#**^. (DOCX 22 kb)

## Data Availability

The data generated and used in the analysis of this study are included in this published article. Additional data is available from the authors upon reasonable request.

## Abbreviations

FA: Folic Acid
KAP: Knowledge, Attitude and Practice
