# Supplementary material for: Knowledge, attitude and practice level of women at the periconceptional period: a cross-sectional study in Shaanxi China
Source: BMC Pregnancy Childbirth. 2019 Sep 4;19:326. doi: 10.1186/s12884-019-2481-6 (PMC6727354; doi:10.1186/s12884-019-2481-6)
Supplement: Supplementary file 1 — Table S1. Birth defects in Xi’an from 2003 to 2016. (DOCX 21 kb) [file 12884_2019_2481_MOESM1_ESM.docx]

Table S1 Birth defects in Xi’an from 2003-2016

| **Year** | **Perinatal**  **infants (N)** | **Birth defects [N (‰)]** | **Neural tube defects** | |  | **Polydactyly** | |  | **Congenital hydrocephalus** | |  | **Cleft lip with cleft palate** | |  | **Cleft lip** | |  | **Congenital heart disease** | |
| --- | --- | --- | --- | --- | --- | --- | --- | --- | --- | --- | --- | --- | --- | --- | --- | --- | --- | --- | --- |
|  |  |  | **N (‰)** | **rank** |  | **N (‰)** | **rank** |  | **N (‰)** | **rank** |  | **N (‰)** | **rank** |  | **N (‰)** | **rank** |  | **N (‰)** | **rank** |
| 2003 | 42272 | 392 (9.27) | 100 (2.366) | 1 |  | 47 (1.112) | 2 |  | 43 (1.017) | 3 |  | 42 (0.994) | 4 |  | 28 (0.662) | 5 |  | 16 (0.379) | - |
| 2004 | 48489 | 429 (8.85) | 97 (2.000) | 1 |  | 62 (1.279) | 2 |  | 48 (0.990) | 3 |  | 46 (0.949) | 4 |  | 29 (0.598) | 5 |  | 13 (0.268) | - |
| 2005 | 54827 | 422 (7.70) | 105 (1.915) | 1 |  | 56 (1.021) | 3 |  | 54 (0.985) | 4 |  | 57 (1.040) | 2 |  | 30 (0.547) | 5 |  | 13 (0.237) | - |
| 2006 | 58653 | 469 (8.00) | 94 (1.603) | 1 |  | 69 (1.176) | 2 |  | 48 (0.818) | 4 |  | 62 (1.057) | 3 |  | 27 (0.460) | - |  | 29 (0.494) | 5 |
| 2007 | 80530 | 601 (7.46) | 96 (1.192) | 1 |  | 90 (1.118) | 2 |  | 51 (0.633) | 4 |  | 86 (1.068) | 3 |  | 46 (0.571) | 5 |  | 36 (0.447) | - |
| 2008 | 96547 | 698 (7.23) | 113 (1.17) | 1 |  | 91 (0.943) | 2 |  | 74 (0.766) | 4 |  | 82 (0.849) | 3 |  | 54 (0.559) | 5 |  | 35 (0.363) | 6 |
| 2009 | 98921 | 843 (8.52) | 124 (1.25) | 2 |  | 136 (1.375) | 1 |  | 32 (0.738) | 4 |  | 90 (0.910) | 3 |  | 61 (0.617) | 5 |  | 55 (0.556) | 6 |
| 2010 | 106822 | 774 (7.25) | 61 (0.571) | 4 |  | 152 (1.423) | 1 |  | 73 (0.300) | 9 |  | 80 (0.749) | 3 |  | 43 (0.402) | 5 |  | 102 (0.955) | 2 |
| 2011 | 113974 | 947 (8.31) | 111 (0.973) | 2 |  | 149 (1.307) | 1 |  | 77 (0.676) | 5 |  | 106 (0.930) | 3 |  | 48 (0.421) | 6 |  | 84 (0.737) | 4 |
| 2012 | 131928 | 981 (7.43) | 124 (0.940) | 3 |  | 148 (1.122) | 2 |  | 93 (0.705) | 4 |  | 77 (0.584) | 5 |  | 47 (0.356) | 6 |  | 159 (1.250) | 1 |
| 2013 | 132088 | 1163 (8.80) | 62 (0.469) | 4 |  | 174 (1.317) | 2 |  | 61 (0.462) | 5 |  | 68 (0.515) | 3 |  | 57 (0.432) | 6 |  | 333 (2.521) | 1 |
| 2014 | 144378 | 1450 (10.04) | 63 (0.436) | 4 |  | 198 (1.371) | 2 |  | 56 (0.388) | 5 |  | 65 (0.450) | 3 |  | 43 (0.298) | 8 |  | 568 (3.934) | 1 |
| 2015 | 127558 | 1477 (11.34) | 32 (0.251) | 3 |  | 97 (0.760) | 2 |  | 32 (0.251) | 3 |  | 13 (0.102) | 10 |  | 15 (0.118) | 9 |  | 430 (3.371) | 1 |
| 2016 | 143449 | 1570 (10.94) | 30 (0.209) | 10 |  | 209 (1.457) | 2 |  | 31 (0.216) | 9 |  | 52 (0.363) | 5 |  | 44 (0.307) | 6 |  | 641 (4.468) | 1 |
