# Supplementary material for: Knowledge, attitude and practice level of women at the periconceptional period: a cross-sectional study in Shaanxi China
Source: BMC Pregnancy Childbirth. 2019 Sep 4;19:326. doi: 10.1186/s12884-019-2481-6 (PMC6727354; doi:10.1186/s12884-019-2481-6)
Supplement: Supplementary file 2 — Table S2. Ten closed choice questions to assess knowledge level section#. (DOCX 22 kb) [file 12884_2019_2481_MOESM2_ESM.docx]

Table S2: 10 closed choice questions to assess knowledge level section**^#^**

|  | **Themes** | **Questions** | **Options** | **Correct answer** |
| --- | --- | --- | --- | --- |
| 1 | The concept of birth defect | What is birth defect？ | a. It is the abnormal development of the fetus in the maternal womb before birth.  b. It is the disease that the mother transmitted to the child.  c. It is the genetic disease.  d. It is the damage to the newborn that is caused during childbirth. | a |
| 2 | Birth defects classification | Please tick the options for birth defects you know. (multiple-choice) | a. Anencephalus  b. Hydrocephalus  c. Cleft lip and palate  d. Congenital heart disease | a,b,c and d* |
| 3 | Primary prevention | What is the concept of primary prevention of birth defects? | a. To prevent birth defects.  b. To reduce the number of babies born with birth defects.  c. To improve the quality of life of children with birth defects.  d. To treat children with birth defects. | a |
| 4 | Neonatal screening | All my examinations during the pregnancy were normal, and the ultrasound examinations were normal as well, so does my child need to have a health check at birth? | a. Yes, needs to be examined.  b. No, because the results of examinations during my pregnancy are all normal.  c. No, because the examinations during pregnancy were already enough.  d. Have no idea. | a |
| 5 | Men’s pre-pregnancy check | Do men need to have a pre-pregnancy examination? | a. No, he has done pre-marital examination and all the results are normal, do not need to do pre-pregnancy examination.  b. No, he is usually in good health, so he doesn't have to do it.  c. Yes, premarital examination can’t replace pre-pregnancy examination.  d. No, it is a woman’s business to give birth to a child, men don't need a pre-pregnancy examination. | c |
| 6 | FA supplementary time | Ordinary women who take folic acid during pregnancy can reduce the risk of neural tube defects in offspring, and how should it be supplemented? | a. Take 0.4 mg of folic acid supplements daily from three months before pregnancy until you are already pregnant.  b. Take 0.4 mg of folic acid supplements per week from three months before pregnancy to the first trimester.  c. Take 0.4 mg of folic acid supplement daily from the beginning of pregnancy to the first trimester.  d. Take 0.4 mg of folic acid supplement daily from three months before pregnancy to the first trimester. | d |
| 7 | FA supplementary dosage | What are the best nutritional supplements for women who are planning to become pregnant after one month to improve maternal and child health and prevent birth defects? | a. Take 0.4mg of folic acid per day.  b. Take a multivitamin with 0.4mg of folic acid daily.  c. Take a multivitamin with 0.8mg of folic acid daily.  d. Take 5mg of folic acid daily. | b |
| 8 | Importance of prenatal examination | All my examinations before pregnancy were normal, do I need to have prenatal examination? | 1. Yes, because the pre-pregnancy examinations can’t take the place of prenatal examinations. 2. No, because many items have been checked before pregnancy, it is enough to guarantee a healthy baby. 3. Not necessarily required, prenatal examination is only necessary if there is a problem in pre-pregnancy check 4. Have no idea. | a |
| 9 | The concept of advanced age pregnancy | How old a pregnant woman over than will be in a high-risk pregnancy? | a. 25 years of age.  b. 30 years of age.  c. 35 years of age.  d. 40 years of age. | c |
| 10 | The association between thyroid dysfunction and eugenics | Is there an association between maternal hypothyroidism and offspring health? | a. Yes, it can affect fetal intelligence and physical development.  b. No, the fetus has thyroid gland, which is not affected by the mother.  c. Have no idea. | a |

^#^: Closed quantify scoring system was used for the 10 questions, where 1 question corresponded to 1 point. Each individual received one point for correct answer of each question. It was defined as knowledge pass when the score was higher than or equal to 6.

*: It was considered as correct if all four options had been selected. Missing or not selected were considered as incorrectly.
